# Seroprevalence of fascioliasis, toxocariasis, strongyloidiasis and cysticercosis in blood samples diagnosed in Medic Medical Center Laboratory, Ho Chi Minh City, Vietnam in 2012

**DOI:** 10.1186/s13071-016-1780-2

**Published:** 2016-09-05

**Authors:** Toan Nguyen, Fei Wen Cheong, Jonathan Wee Kent Liew, Yee Ling Lau

**Affiliations:** 1 Medic Medical Center Laboratory, Ho Chi Minh City, Vietnam; 2 Department of Parasitology, Tropical Infectious Diseases Research and Education Center (TIDREC), Faculty of Medicine, University of Malaya, Kuala Lumpur, Malaysia

**Keywords:** Seroprevalence, Fascioliasis, Toxocariasis, Strongyloidiasis, Cysticercosis, Southeast Asia

## Abstract

**Background:**

Despite the global effort against neglected tropical diseases (NTDs), developing countries with middle to low income are still burdened by them. Vietnam has been undergoing substantial economic growth and urbanization, but underprivileged people living in rural and suburban areas are still having little access to public health infrastructure and proper sanitation. Hitherto, limited information is available for seroprevalence and risk factors of several parasitic diseases in Vietnam.

**Methods:**

A retrospective study was performed on diagnostic results of *Fasciola* spp., *Toxocara* spp., *Strongyloides stercoralis* and *Taenia solium* IgG ELISA tests from Medic Medical Center Laboratory, Ho Chi Minh City in 2012. The data were first stratified before statistical analyses were performed. Seroprevalence of fascioliasis, toxocariasis, strongyloidiasis and cysticercosis was determined and the age and gender risk factors were evaluated.

**Results:**

Seroprevalence of fascioliasis, toxocariasis, strongyloidiasis and cysticercosis was 5.9 % (590/10,084; 95 % CI: 5.44–6.36), 45.2 % (34,995/77,356; 95 % CI: 44.85–45.55), 7.4 % (3,174/42,920; 95 % CI: 7.15–7.65) and 4.9 % (713/14,601; 95 % CI: 4.55–5.25), respectively. Co-exposure to multiple parasites was detected in 890 males (45.7 %; 95 % CI: 43.49–47.91) and 1,059 females (54.3 %; 95 % CI: 52.09–56.51). Social structure and differences in behavioural factors caused the gender factor to have a significant effect on the prevalence of all the diseases, while the seropositivity for fascioliasis and strongyloidiasis were age group-related.

**Conclusions:**

The seroprevalence of fascioliasis, toxocariasis, strongyloidiasis and cysticercosis in the blood samples diagnosed in Medic Medical Center Laboratory, Ho Chi Minh City, in year 2012 were comparatively high. The Vietnamese customs and cultures, dietary habits and agricultural practices exposed them to high risk of contracting NTDs. Despite the possibility of false positive results due to antigenic cross-reactions, detection of IgG antibodies remains as a reliable method in sero-epidemiological study as it is non-invasive and demonstrates previous exposure of individuals to the parasites. Besides the implementation of strategies to control these diseases, epidemiological analysis and surveillance of diseases should also be continually strengthened to monitor the effectiveness of regimens and interventions.

## Background

The global fight against neglected tropical diseases (NTDs) has continued to intensify with the adoption of the United Nations Sustainable Development Goals (SDGs) in 2015 [1]. These Global Goals seek, among others, to ensure healthy lives and access to water and sanitation for everyone, regardless of age. With these goals, we may only be one step away from the elimination of NTDs, which would then pave the way for the eradication of extreme global poverty, another goal of the SDGs. Neglected tropical diseases have always been correlated with poverty. While they cause morbidities worldwide, countries of low to middle income, where people live in poverty and have little access to sanitation, clean water and public healthcare, are most heavily burdened by these diseases.

Over the past two decades, Vietnam has made sound progress in the control of lymphatic filariasis and trachoma, besides achieving the World Health Organization (WHO) target of deworming 75 % of school-aged children [2]. Nevertheless, more still needs to be done. The country has been experiencing remarkable economic growth and progress towards reducing poverty [3]. However, people from the rural areas and deprived inhabitants of the suburbs and peri-urban areas remain poor. Furthermore, most Vietnamese are employed in the agricultural, manufacturing, food processing and service sectors. Adding to that, the locals’ customs, eating habits, agricultural practices and activities put them at a high risk of contracting NTDs [4]. In 2011, 148 outbreaks with 38,915 cases and 27 deaths due to food-borne disease were reported in Vietnam [5].

Various strategies have been put into place to control food-borne trematodiases, soil transmitted helminthiasis and parasitic zoonosis in the country. Continuous disease surveillance is therefore needed to track treatment impact and provide information on disease prevalence. Hitherto, several NTDs such as fascioliasis (caused by the liver flukes *Fasciola* spp.), taeniasis (caused by adult tapeworms *Taenia* spp.) and cysticercosis (caused by larvae of *Taenia solium*) are relatively well studied but there has been a lack of knowledge about the prevalence rates of other medically important parasitic diseases in Vietnam, especially toxocariasis (caused by the roundworms *Toxocara* spp.) and strongyloidiasis (caused by the threadworm *Strongyloides stercoralis*). In 2011, more than 20,000 cases of fascioliasis were reported in the central region of Vietnam [6, 7] while 229 out of 1,524 patients had cysticercosis, as reported by the National Institute of Malariology, Parasitology and Entomology (NIMPE) Clinic in 2010 [8]. Meanwhile, *Toxocara* spp. infection rates were reported to be 32.5 % in the southern, 30.2 % in the middle and 33.3 % in the northern parts of Vietnam [9] whereas a 5 % serological infection rate of *S. stercoralis* was detected in the Phu Cat district, Binh Dinh province in 2009 [10]. Information on infection rates and risks in specific groups of the population is clinically and epidemiologically important. Thus, we have retrospectively investigated the seroprevalence of fascioliasis, toxocariasis, strongyloidiasis and cysticercosis in blood samples tested in a diagnostic centre in the urban metropolis of Ho Chi Minh (HCMC), Vietnam, in the year 2012.

## Methods

### Study setting

The retrospective study was performed on diagnostic results of *Fasciola* spp., *Toxocara* spp*.*, *Strongyloides stercoralis* and *Taenia solium* IgG ELISA tests from Medic Medical Center laboratory, HCMC, Vietnam in the year 2012. Medic Medical Center Laboratory is a reference diagnostic lab which receives blood samples from around the country, mostly from the city and the Mekong Delta region which lies west of the city. In year 2012, the average population number in HCMC and the Mekong Delta region was 7.66 million and 17.38 million, respectively [11]. Diagnostic tests were performed as requested by physicians albeit, a lesser number, by the patients who visited the lab.

Data were collected and analyzed anonymously. The anonymized data include all patients and individuals who took the tests from January to December 2012. Information collected consists of age, date of serum collection, gender and result of test. A total number of 10,084, 77,356, 42,920 and 14,601 individuals were tested with the *Fasciola* spp., *Toxocara* spp*.*, *Strongyloides stercoralis* and *Taenia solium* IgG ELISA tests, respectively. Participants were categorized into groups according to gender (male/female) and age group (< 1, 1–15, 16–30, 31–45, 46–60, 61–75, 76–90, > 90 year-old).

### Laboratory procedures

All tests were performed by Medic Medical Center Laboratory using IgG ELISA kits according to the manufacturer’s instructions. In all tests, absorbance was measured bichromatically at 450/620–650 nm. The *Fasciola* spp. IgG ELISA kit (DRG Instruments GmbH, Marburg, Germany) utilizes immobilized *Fasciola* spp. antigen from adult liver fluke. Samples were considered positive if results showed a DU (DRG unit) of > 11. The manufacturer reported a diagnostic sensitivity and specificity of 100 %. The *Toxocara* spp. IgG ELISA kit (DRG Instruments GmbH, Springfield, USA) has the *Toxocara* spp. excretory antigen as the solid phase antigen. Positive samples show ≥ 0.3 OD reading. The kit shows a concordance of 84 %, sensitivity of 87.5 % and specificity of 93.3 %. The *Strongyloides stercoralis* IgG ELISA kit (DRG Instruments GmbH, Springfield, USA) uses microwells coated with *Strongyloides* L3 antigen. Samples were considered positive if results showed absorbance reading ≥ 0.2 OD. The kit’s performance is of 100 % for both sensitivity and specificity. For serodiagnosis of cysticercosis, the *Taenia solium* IgG ELISA kit (DRG Instruments GmbH, Springfield, USA) utilizes *T. solium* cyst fluid antigen for qualitative screening of serum IgG to *T. solium.* Samples with absorbance reading ≥ 0.3 OD were considered positive. This test has a reported sensitivity of 87–88 % and specificity of 96 %.

### Data analysis

Statistical analyses was performed in the Department of Parasitology, University of Malaya, Malaysia using statistical package SPSS v.20 (SPSS IBM, US). Prior to analyses, the data were stratified according to gender (male/female) and age group in years (≤ 60/> 60). Pearson’s Chi-square test was used in bivariate analysis to determine the association of disease seroprevalence with age and gender. Binary logistic regression analysis was performed in multivariable analysis to identify the significant risk factor variables and to control the confounders. Relative risk (RR), odds ratio (OR) and 95 % confidence interval (95 % CI) were calculated, and the statistical significance level for all tests was set at *P* < 0.05.

## Results

The descriptive characteristics of participants according to gender and age group for fascioliasis, toxocariasis, strongyloidiasis and cysticercosis are presented in Table 1.

**Table 1:** Descriptive characteristics of the participants

Characteristics	Fascioliasis (*n* = 10,084)	Toxocariasis (*n* = 77,356)	Strongyloidiasis (*n* = 42,920)	Cysticercosis (*n* = 14,601)
	*n* (%)	*n* (%)	*n* (%)	*n* (%)
Gender				
Male	4,916 (48.8)	30,698 (39.7)	16,651 (38.8)	5,889 (40.3)
Female	5,168 (51.2)	46,658 (60.3)	26,269 (61.2)	8,712 (59.7)
Age group (years)				
< 1	22 (0.2)	120 (0.2)	71 (0.2)	27 (0.2)
1–15	728 (7.2)	12,007 (15.5)	5,924 (13.8)	1,750 (12.0)
16–30	2,151 (21.3)	19,842 (25.7)	10,529 (24.5)	3,482 (23.8)
31–45	3,823 (37.9)	26,488 (34.2)	14,681 (34.2)	5,328 (36.5)
46–60	2,718 (27.0)	15,342 (19.8)	9,048 (21.1)	3,132 (21.5)
61–75	494 (4.9)	2,908 (3.8)	2,089 (4.9)	695 (4.8)
76–90	140 (1.4)	627 (0.8)	545 (1.3)	175 (1.2)
> 90	8 (1.0)	22 (< 0.1)	33 (0.1)	12 (0.1)

### Seroprevalence of fascioliasis

Out of the 10,084 serum samples tested, 590 (5.9 %; 95 % CI: 5.44–6.36) were positive for *Fasciola* spp. antibodies. Bivariate analysis was carried out to determine the association between seroprevalence with gender and age group (Table 2). Both variables were found to have statistically significant association with seroprevalence of fascioliasis. Lower prevalence was found in males compared to females (RR: 0.817; 95 % CI: 0.70–0.96), and age group of ≤ 60 year-old exhibited a lower risk than age group of > 60 year-old (RR: 0.719; 95 % CI: 0.55–0.95). Multivariable logistic regression analysis (Table 3) detected the same pattern of association as bivariate analysis, where both risk factors were identified as significant predictors.

**Table 2:** Univariate and bivariate analysis for the association between seroprevalence of diseases and risk factors

Risk factor	Fascioliasis	Toxocariasis	Strongyloidiasis	Cysticercosis
	*n*/total *n* (%)	*n*/total *n* (%)	*n*/total *n* (%)	*n*/total *n* (%)
Gender				
Male	258/4,916 (5.2)	14,533/30,698 (47.3)	1,429/16,651 (8.6)	330/5,889 (5.6)
Female	332/5,168 (6.4)	20,462/46,658 (43.9)	1,745/26,269 (6.6)	383/8,712 (4.4)
RR	0.817	1.080	1.292	1.275
(95 % CI)	(0.70–0.96)	(1.06–1.10)	(1.21–1.38)	(1.10–1.47)
*P*-value	0.012	< 0.0001	< 0.0001	0.001
Age group (years)				
≤ 60	539/9,442 (5.7)	33,363/73,799 (45.2)	2,874/40,253 (7.1)	659/13,719 (4.8)
> 60	51/642 (7.9)	1,632/3,557 (45.9)	300/2,667 (11.2)	54/882 (6.1)
RR	0.719	0.985	0.635	0.785
(95 % CI)	(0.55–0.95)	(0.95–1.02)	(0.57–0.71)	(0.60–1.03)
*P*-value	0.020	0.431	< 0.0001	0.078

**Table 3:** Multivariable analysis for the association between seroprevalence of diseases and risk factors

Risk factor	Fascioliasis	Toxocariasis	Strongyloidiasis	Cysticercosis
	OR (95 % CI)	OR (95 % CI)	OR (95 % CI)	OR (95 % CI)
Gender				
Male/female	0.814 (0.69–0.96)	1.152 (1.12–1.19)	1.330 (1.24–1.43)	1.297 (1.12–1.51)
*P*-value	0.016	< 0.0001	< 0.0001	0.001
Age group (years)				
≤ 60/> 60	0.715 (0.53–0.96)	0.964 (0.90–1.03)	0.597 (0.53–0.68)	0.761 (0.57–1.01)
*P*-value	0.028	0.283	< 0.0001	0.062

### Seroprevalence of toxocariasis

A total of 34,995 out of 77,356 serum samples (45.2 %; 95 % CI: 44.85–45.55) were positive for *Toxocara* spp. antibodies. Bivariate analysis showed that gender was significantly associated with the seropositivity of toxocariasis (Table 2). Prevalence in males was slightly higher than in females, with a RR of 1.080 (95 % CI: 1.06–1.10). Binary logistic regression also yielded the same pattern of results, indicating that seroprevalence was significantly associated with gender but not age group (Table 3).

### Seroprevalence of strongyloidiasis

*Strongyloides stercoralis* antibodies were detected in 3,174 of the 42,920 samples tested (7.4 %; 95 % CI: 7.15–7.65). Both univariate and multivariable analysis demonstrated that gender and age group were both significant risk factors for seroprevalence of strongyloidiasis (Tables 2 and 3). Results indicated that males had a higher risk of *Strongyloides stercoralis* infection than females (RR: 1.292; 95 % CI: 1.21–1.38), while lower risk of infection was observed in age group of ≤ 60 year-old than age group of > 60 year-old (RR: 0.635; 95 % CI: 0.57–0.71).

### Seroprevalence of cysticercosis

Of the 14,601 serum samples, *Taenia solium* antibodies were detected in 713 samples (4.9 %; 95 % CI: 4.55–5.25). Gender was the only statistically significant variable associated with cysticercosis in both Pearson’s Chi-square test (Table 2) and binary logistic regression analysis (Table 3). Of that, higher seroprevalence of cysticercosis was found in males (RR: 1.275; 95 % CI: 1.10–1.47), whereas age group of ≤ 60 year-old had a lower risk of infection than age group of > 60 year-old (RR: 0.785; 95 % CI: 0.60–1.03).

### Co-exposure rate

Table 4 summarizes the seroprevalence of co-exposure to fascioliasis, toxocariasis, strongyloidiasis and cysticercosis. Co-exposure to multiple parasites was detected in 890 males (45.7 %; 95 % CI: 43.49–47.91) and 1,059 females (54.3 %; 95 % CI: 52.09–56.51). Among them, co-exposure to strongyloidiasis and toxocariasis was the most prevalent (1,362/1,949, 69.9 %; 95 % CI: 67.86–71.94), followed by that of cysticercosis and toxocariasis (211/1,949, 10.8 %; 95 % CI: 9.42–12.18) and cysticercosis and strongyloidiasis (110/1,949, 5.6 %; 95 % CI: 4.58–6.62). Five persons (0.3 %; 95 % CI: 0.06–0.54) were found to be seropositive for antibodies against all of the four helminths. A total of 36.8 %, 26.4 % and 17.9 % of the co-exposures occurred in 31–45 year-old (718/1,949; 95 % CI: 34.66–38.94), 46–60 year-old (515/1,949; 95 % CI: 24.44–28.36) and 16–30 year-old (348/1,949; 95 % CI: 16.20–19.60) patients, respectively.

**Table 4:** Seroprevalence of co-exposure to fascioliasis, toxocariasis, strongyloidiasis and cysticercosis

Risk factor		F + S	F + T	C + T	S + T	C + S	C + F	F + S + T	F + S + C	C + T + S	C + T + F	F + S + C + T	Total
Gender	Age group	*n* (%)	*n* (%)	*n* (%)	*n* (%)	*n* (%)	*n* (%)	*n* (%)	*n* (%)	*n* (%)	*n* (%)	*n* (%)	*n* (%)
Male	< 1	1 (3.7)	0 (0.0)	0 (0.0)	1 (0.1)	1 (0.9)	0 (0.0)	0 (0.0)	0 (0.0)	0 (0.0)	0 (0.0)	0 (0.0)	3
	1–15	2 (7.4)	2 (1.9)	52 (24.6)	46 (3.4)	8 (7.3)	0 (0.0)	0 (0.0)	0 (0.0)	6 (5.7)	0 (0.0)	0 (0.0)	116
	16–30	1 (3.7)	6 (5.6)	17 (8.1)	106 (7.8)	6 (5.5)	1 (33.3)	3 (20.0)	0 (0.0)	9 (8.5)	0 (0.0)	0 (0.0)	149
	31–45	6 (22.2)	23 (21.5)	23 (10.9)	232 (17.0)	14 (12.7)	0 (0.0)	1 (6.7)	0 (0.0)	16 (15.1)	1 (100.0)	0 (0.0)	316
	46–60	2 (7.4)	17 (15.9)	10 (4.7)	163 (12.0)	15 (13.6)	0 (0.0)	2 (13.3)	1 (50.0)	7 (6.6)	0 (0.0)	1 (20.0)	218
	61–75	0 (0.0)	3 (2.8)	2 (0.9)	51 (3.7)	5 (4.5)	0 (0.0)	0 (0.0)	0 (0.0)	7 (6.6)	0 (0.0)	1 (20.0)	69
	76–90	0 (0.0)	0 (0.0)	1 (0.5)	17 (1.2)	0 (0.0)	0 (0.0)	0 (0.0)	0 (0.0)	1 (0.9)	0 (0.0)	0 (0.0)	19
	> 90	0 (0.0)	0 (0.0)	0 (0.0)	0 (0.0)	0 (0.0)	0 (0.0)	0 (0.0)	0 (0.0)	0 (0.0)	0 (0.0)	0 (0.0)	0
Female	< 1	0 (0.0)	0 (0.0)	0 (0.0)	2 (0.1)	0 (0.0)	0 (0.0)	0 (0.0)	0 (0.0)	0 (0.0)	0 (0.0)	0 (0.0)	2
	1–15	1 (3.7)	1 (0.9)	14 (6.6)	50 (3.7)	3 (2.7)	1 (33.3)	1 (6.7)	0 (0.0)	2 (1.9)	0 (0.0)	0 (0.0)	73
	16–30	5 (18.5)	13 (12.1)	21 (10.0)	141 (10.4)	9 (8.2)	0 (0.0)	2 (13.3)	0 (0.0)	8 (7.5)	0 (0.0)	0 (0.0)	199
	31–45	7 (25.9)	22 (20.6)	40 (19.0)	284 (20.9)	19 (17.3)	1 (33.3)	2 (13.3)	1 (50.0)	26 (24.5)	0 (0.0)	0 (0.0)	402
	46–60	1 (3.7)	14 (13.1)	29 (13.7)	210 (15.4)	26 (23.6)	0 (0.0)	2 (13.3)	0 (0.0)	13 (12.3)	0 (0.0)	2 (40.0)	297
	61–75	0 (0.0)	5 (4.7)	1 (0.5)	48 (3.5)	2 (1.8)	0 (0.0)	2 (13.3)	0 (0.0)	9 (8.5)	0 (0.0)	1 (20.0)	68
	76–90	1 (3.7)	1 (0.9)	1 (0.5)	9 (0.7)	2 (1.8)	0 (0.0)	0 (0.0)	0 (0.0)	2 (1.9)	0 (0.0)	0 (0.0)	16
	> 90	0 (0.0)	0 (0.0)	0 (0.0)	2 (0.1)	0 (0.0)	0 (0.0)	0 (0.0)	0 (0.0)	0 (0.0)	0 (0.0)	0 (0.0)	2
Total		27	107	211	1,362	110	3	15	2	106	1	5	1,949

*Abbreviations: F* fascioliasis, *T* toxocariasis, *S* strongyloidiasis, *C* cysticercosis

## Discussion

Little information is available on the prevalence of parasitic infections in Vietnam especially on toxocariasis and strongyloidiasis. The seroprevalence rates found in this study may reflect that of people living in the southwest region of Vietnam, including HCMC, as most of the blood samples received originated from this region. Seroprevalence rates for all infections reported here are high, especially for toxocariasis (45.2 %) and strongyloidiasis (7.4 %). Transmission of some of the diseases can be explained by the dietary habits of the locals. For example, consuming raw vegetables such as kangkong-kalabau (*Enhydra fluctuants*) and Brahmi (*Herpestis monniera*) contaminated with metacercariae of *Fasciola* spp., or eating raw/pickled pork (nem chua and nem chao), vegetables and dog meat contaminated with taeniid eggs and cysts of *T. solium* [12, 13]. Besides, proximity with infected livestock, use of human faeces as fertilizer, poor sanitation and others, further increase disease transmission to humans [5, 14, 15].

In Vietnam, fascioliasis is caused by *Fasciola gigantica* and the hybrid of *Fasciola hepatica* and *Fasciola gigantica* [16]. This emerging food-borne trematodiasis [5, 17] is transmitted in most of the provinces in Vietnam, particularly in the central regions [18] with an overall anti-*Fasciola* spp*.* IgG seroprevalence of 7.8 % among adult cohorts in two central provinces of Vietnam in 2013 [19]. In this study, a 5.9 % seroprevalence of fascioliasis was detected, which is in accordance with the World Health Organization [20] report stating that prevalence can reach a high 5.4 % at endemic foci. This suggests that not only the central region, but other regions of the country may pose a high seroprevalence of fascioliasis too. In endemic foci, infected patients are usually women aged between 17 and 45 years; also in some areas, the number of infected women are about three times the number of infected men [6, 20, 21]. In our study, females aged between 16 and 45 years constituted one third of the total exposure rate (31.7 %). Females may face a higher exposure rate to water and freshwater plants that carry metacercariae, as they are responsible for household chores.

Toxocariasis is commonly transmitted by accidental swallowing of soil contaminated with *Toxocara* spp.eggs. In the present study, a seroprevalence of 45.2 % was detected for toxocariasis, higher than the reported infection rates of 30.2–33.3 % in parts of Vietnam [9]. Seroprevalence of toxocariasis in Vietnam appears to be one of the highest in the world when compared with that of other countries [22–26]. Contacts with *Toxocara*-infected cats and dogs is one of the main factors for human toxocariasis transmission. A recent study in 2014 showed high toxocariasis prevalence in household cats (47.8 %) and dogs (37.7 %) in Hanoi city, Vietnam, which partly contributed to the finding of anti-*Toxocara* IgG antibodies in 58.7 % of the human samples. Moreover, the high egg excretion rates of infected dogs or cats, along with the large numbers of free roaming stray animals increase contamination of soil and vegetables with *Toxocara* spp. eggs [27], leading to higher transmission rate. Males were found to have greater exposure to toxocariasis than females, similar to other findings [26, 28–31]. Previous studies also found that prevalence of toxocariasis is significantly higher in children due to their geophagia, playing and social behaviours, which is in disagreement with our finding and another study [25] that age group is not a significant association factor. Deworming household pets, proper hygiene, adequate cooking of food and education to the public [32] are some measures that can be taken to reduce the high levels of *Toxocara* spp. infection in the country.

Infection with *Strongyloides stercoralis* is endemic in many tropical and subtropical countries [33]. Even so, there is limited data on infection rates of *S. stercoralis* in Vietnam. Our study revealed 7.4 % seropositivity for strongyloidiasis, which is slightly lower than that in the neighbouring southeast Asian countries of Cambodia (17.5 %), Lao PDR (26.2 %), and Thailand (23.7 %) [34]. A prevalence study in Vietnam had detected a 5 % serological infection rate of *S. stercoralis* in the Phu Cat district, Binh Dinh province in 2009 [10]. Humans become infected through contact with *Strongyloides* larvae-contaminated soil, while playing or working barefoot on the ground. Similar to other studies conducted in Thailand [35] and Cambodia [36], a higher rate of *S. stercoralis* infection in men than in women was found in this study. This is most likely due to males working outdoors, barefooted while the females usually work at home and have the habit of wearing shoes when walking on the ground [35].

Cysticercosis develops in human hosts who have ingested soil, water or food contaminated with the eggs of the pork tapeworm *Taenia solium* [5]. The 2014 prevalence of cysticercosis in northern and southern Vietnam was 1.0–7.2 % and 4.3 %, respectively [8]. The 4.9 % prevalence of cysticercosis found in the present study is in agreement with the above reports. The gender risk factor for cysticercosis infection could be explained by the social structure of Vietnam. In Vietnam, males have the primary role of supporting and sustaining the family by working outdoors, increasing their exposure rate to *Taenia*-contaminated sources. Willingham et al. [37] reported that 70 % of cysticercosis patients seen at hospitals were male. As in the present study, several seroprevalence studies have also revealed that seropositivity for cysticercosis is not significantly related to age group [38–41].

In our study, 1,949 individuals from among those seropositive for the tested parasites were also positive for more than one parasite. In fact, in 2011, more than 60 % of infected cases in Vietnam were found to have two to five kinds of parasites [7]. Studies done in Southern Laos [42] and on urban dwellers in Ethiopia [43] show that polyparasitism is very common (52.4 % and 56.7 %, respectively). It was found that co-exposure to toxocariasis and strongyloidiasis was the most prevalent. It is possibly due to the highest prevalence of the two diseases among the four NTDs, shared routes of transmission such as soil and the parasites co-existence in the environment. However, the co-exposure rates should also be interpreted with care. It has been evident that cross-reactions often happen in ELISA-based serological tests, leading to false positive results. Cross-reactions with *Strongyloides*, *Fasciola*, *Ascaris*, *Schistosoma* spp. and other parasites were reported when excretory/secretory (E/S) antigen from second-stage larvae of *Toxocara canis* was used in ELISA [44, 45]. Use of *S. stercoralis* filariform/L3 larvae antigen in ELISA was shown to be ≥ 88 % sensitive and ≥ 96.1 % specific in detecting specific anti-IgG, with possible cross-reaction with toxocariasis, *Ascaris lumbricoides* and others [46, 47].

A positive IgG serodiagnosis can be caused by any residual antibodies after successful treatment or an ongoing, chronic infection. However, ELISA-based serological tests rather than parasitological methods are preferred due to the better specificity and sensitivity [6]. Serological test to detect specific IgG, not IgM or IgA, is recommended for most parasitic diseases [48]. Therefore, detection of IgG antibodies in this sero-epidemiological study is deemed appropriate and demonstrates exposure of individuals to the parasites. Nonetheless, the authors agree that accurate diagnosis should be done in conjunction with other clinical findings, epidemiological factors and other laboratory results. The current study would benefit from stool studies to determine active infection, complementing the serological test results.

## Conclusions

The overall IgG seroprevalence rates against the parasites were found to be relatively high (fascioliasis, 5.9 %; toxocariasis, 45.2 %; strongyloidiasis, 7.4 %; cysticercosis, 4.9 %) among individuals diagnosed in the Medic Medical Center Laboratory, Ho Chi Minh City throughout the year 2012. Analyses revealed that females are more exposed to *Fasciola* spp. while men have significant risks of toxocariasis, strongyloidiasis and cysticercosis. Furthermore, people older than 60 years showed a significant higher risk of being infected with fascioliasis and strongyloidiasis. The four parasitic diseases investigated in this retrospective study are environment-related parasitic infections. Therefore, proper hygiene and sanitation must be observed in order to control the diseases. The current study also highlighted epidemiological data of strongylodiasis and toxocariasis in Vietnam, which are scarce in the region. Continual epidemiological analysis and food-borne zoonosis surveillance should be carried out in order to monitor the effectiveness of the regimens employed and also to revamp or implement more successful interventions.

## Abbreviations

CI: Confidence interval
DU: DRG units
ELISA: Enzyme-linked immunosorbent assay
HCMC: Ho Chi Minh City
Ig: Immunoglobulin
NIMPE: National Institute of Malariology, Parasitology and Entomology
NTDs: Neglected tropical diseases
OR: Odds ratio
RR: Relative risk
SDGs: United Nations Sustainable Development Goals
WHO: World Health Organization
